# Suprascapular neuropathy in the setting of rotator cuff tears: study protocol for a double-blinded randomized controlled trial

**DOI:** 10.1186/s13063-016-1672-y

**Published:** 2016-11-22

**Authors:** Nikolaos Platon Sachinis, Achilleas Boutsiadis, Sotirios Papagiannopoulos, Konstantinos Ditsios, Anastasios Christodoulou, Pericles Papadopoulos

**Affiliations:** 1 First Orthopaedic Department, Aristotle University of Thessaloniki, Thessaloniki, Greece; 2 Third Neurology Department, Aristotle University of Thessaloniki, Thessaloniki, Greece; 3 Palaistras 25, Ammoxori, Florina, 53100 Greece

**Keywords:** Rotator cuff tear, Suprascapular neuropathy, Arthroscopy, Superior transverse scapular ligament release, Massive

## Abstract

**Background:**

It has been indicated that rotator cuff tears, especially large or massive ones, can cause suprascapular neuropathy. When such a diagnosis has been established, it is still unknown whether an arthroscopic release of the superior transverse scapular ligament during cuff repair can change the course of this neuropathy.

**Methods/design:**

This is a single-center, double-blinded randomized controlled trial for which 42 patients with large or massive repairable rotator cuff tears and suprascapular neuropathy will be recruited and followed up at 6 and 12 months. Nerve function will be measured by nerve conduction and electromyography studies preoperatively and at the selected follow-up periods. Patients will be randomly divided into equally numbered groups, the first one being the control group. Patients of this group will undergo arthroscopic repair of the rotator cuff without combined arthroscopic release of the superior transverse scapular ligament; in the second group the ligament will be released. The primary objective is to test the null hypothesis that arthroscopic repair of large/massive rotator cuff tears in patients with combined suprascapular neuropathy provides equivalent outcomes to one-stage arthroscopic cuff repair where the superior suprascapular ligament is additionally released. The secondary objective is to search for a relation between rotator cuff tear size and degree of suprascapular nerve recovery. The tertiary objective is to demonstrate any relation between rotator cuff muscle fatty infiltration grade and degree of suprascapular nerve function. Patients, clinicians during follow-up clinics and the neurologist will be blinded to the type of surgery performed.

**Discussion:**

To the best of our knowledge, we are unaware of any prospective, randomized double-blinded studies with similar objectives. So far, the evidence suggests a positive correlation between massive rotator cuff tears and suprascapular neuropathy. However, there is mixed evidence suggesting that neuropathy can be effectively treated with rotator cuff repair with or without release of the superior transverse scapular ligament.

**Trial registration:**

ClinicalTrials.gov registration number NCT02318381; date of initial release: 5 December 2014.

**Electronic supplementary material:**

The online version of this article (doi:10.1186/s13063-016-1672-y) contains supplementary material, which is available to authorized users.

## Background

Suprascapular nerve (SSN) neuropathy may be associated with a rotator cuff tear [1], especially tears bigger than 3 cm. Cofield classified rotator cuff tears as: small tear <1 cm, medium 1–3 cm, large 3–5 cm, and massive >5 cm [2]. The proposed mechanism involves nerve traction that is caused by tethering of the nerve against fixed location points; these are the major and minor scapular notches [3]. Radiographic methods, especially computed tomography (CT) and magnetic resonance imaging (MRI), are often used to evaluate atrophy of the rotator cuff. Fatty infiltration and atrophy may both be associated with a torn rotator cuff and SSN neuropathy [4–6].

Previous studies have reported a relation between SSN neuropathy and rotator cuff tears [7–10]. If these findings are proven accurate, they may represent an important step in optimizing treatment for rotator cuff tears. Nondiagnosed SSN neuropathy could affect the success rate repair of the cuff and further weaken the muscles innervated by this nerve. Theoretically, this neuropathy may be a contributing factor in the pathophysiological mechanism of fatty infiltration and, finally, cuff tear arthropathy although a linear relationship between the degree of neuropathy and the size of the rotator cuff tear has not been demonstrated [9].

Surgical techniques of treating SSN neuropathy in the setting of rotator cuff tears may include open or arthroscopic release nerve release. Open nerve release has provided good results in patients with SSN neuropathy by improving motor function of the rotator cuff muscles [11]. However, arthroscopic release techniques are currently gaining popularity as they can be combined with repair of the torn rotator cuff. They are also less invasive, provide better visualization and require less surgical time [1].

We searched the websites of online trial registries, “http://www.clinicaltrials.gov/,” “http://www.isrctn.com,” “http://onlinelibrary.wiley.com/cochranelibrary,” as well as “http://www.ncbi.nlm.nih.gov/pubmed.” To the best of our knowledge, there are no randomized prospective studies that compare the outcome of SSN neuropathy after arthroscopic dissection of the transverse scapular ligament in patients with a torn rotator cuff against control groups that did not undergo ligament dissection. We are also unaware of any studies that aimed to find any relation between the degree of neuropathy and the size of the rotator cuff tear. The primary objective is to test the null hypothesis that arthroscopic repair of large/massive rotator cuff tears in patients with combined suprascapular neuropathy provides equivalent outcomes to one-stage arthroscopic cuff repair where the superior suprascapular ligament is additionally released. The secondary objective is to search for a relation between rotator cuff tear size and degree of SSN recovery. The tertiary objective is to demonstrate any relation between rotator cuff muscle fatty infiltration grade and degree of SSN function.

## Methods/design

**Fig. 1:**
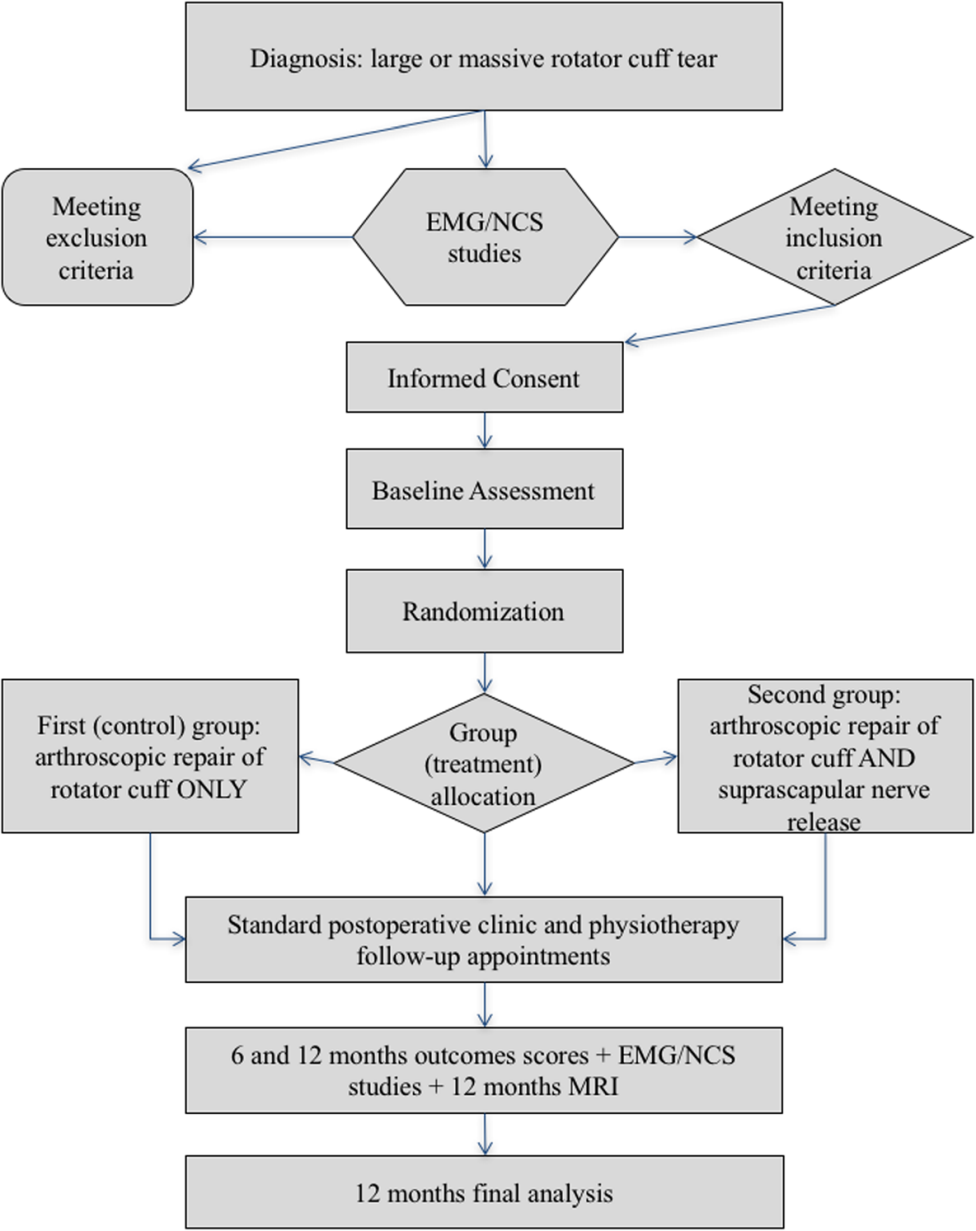
Study flowchart

This study will be a single-center, double-blinded randomized controlled trial (RCT). The trial is registered at the ClinicalTrial.gov website, number NCT02318381. The Institutional Review Board of “Georgios Papanikolaou” Hospital has provided ethical approval. The study will be conducted in accordance with the Helsinki Declaration. All patients will be provided with a written Consent Form that they will have to sign in order to be considered for this study. The study’s Consolidated Standards of Reporting Trials (CONSORT) flowchart and Standard Protocol Items: Recommendations For Interventional Trials (SPIRIT) figure can be found in Fig. 1 and Table 1, respectively. The SPIRIT checklist has been added as Additional file 1 to this protocol.

**Table 1:** Standard Protocol Items: Recommendations for Interventional Trials (SPIRIT) diagram

	Study period					
	Enrollment	Allocation	Post allocation			
Time point	Preintervention	Time 0	Baseline (Intervention)	Post intervention	6-month follow-up	12-month follow-up
Enrollment:						
Eligibility screen	X					
Informed consent	X					
Allocation		X				
Interventions:						
Control group: repair of rotator cuff *only*			X			
Second group: repair of rotator cuff and SSN release			X			
Assessments:						
NC/EMG studies	X				X	X
Clinical shoulder assessment/signs	X			X	X	X
Constant-Murley score	X				X	X
DASH score	X				X	X
ASES score	X				X	X
MRI scan	X					X

*ASES* American Shoulder and Elbow Surgeons, *DASH* Disabilities of the Arm, Shoulder and Hand, *EMG* electromyography, *MRI* magnetic resonance imaging, *NC* nerve conduction, *SSN* suprascapular nerve

### Trial objectives/endpoints

The primary endpoint of this study will be recording of changes of function of the SSN, as assessed by electromyography (EMG) and/or nerve conduction (NC) studies, preoperatively, and at 6 and 12 months postoperatively. All NC/EMG studies, pre and postoperative, will be performed by one neurologist for all included patients. Clinical signs of full recovery will also be assessed and compared pre and postoperatively.

Abnormal function of the SSN in EMG studies will be indicated by fibrillation and high-frequency discharge potentials. An abnormal NC assessment finding is defined according to the following values: infraspinatus latency >4.5 ms and an amplitude <8 mV from peak to peak and supraspinatus muscle latency >3.5 ms and an amplitude <8 mV from peak to peak [12]. The contralateral nerve will also be examined. Abnormal findings are also indicated when the difference in amplitude compared to the healthy side is more than 50%. According to patients’ results, the NC/EMG studies postoperatively will show full recovery of the nerve if all parameters are normal and partial recovery if the tested parameters are improved but not normalized. It is possible, due to some patients’ habitus and size, that NC studies may not be feasible to perform on these patients. In these cases, diagnosis will be established by EMG studies.

The secondary endpoint will be analyzing for possible correlations between tear size, SSN neuropathy frequency, and degree of postoperative nerve recovery. The diameter of the tear will be measured preoperatively with a ruler by using standardized MRI sagittal images. Measurement starts at the insertion of the tendon into the major tuberosity and ends at the most lateral margin of the torn rotator cuff. Only the largest measured diameter of the tear as found on images will be recorded.

The tertiary endpoint will be analyzing the rotator cuff muscles for changes of fatty infiltration as assessed using MRI studies (according to Goutallier’s classification) before and 12 months after the operation [13]. Goutallier classified fatty infiltration as: stage 0, normal muscle; stage 1, some fatty streaks; stage 2, less than 50% fatty muscle atrophy; stage 3, 50% fatty muscle atrophy; stage 4, greater than 50% fatty muscle atrophy.

### Study population

Forty-two patients with SSN neuropathy and repairable large or massive rotator cuff tears will be recruited according to the inclusion/exclusion criteria of this RCT, divided in two equally numbered groups and treated within a 24-month period at the First Orthopedic Department of Aristotle University of Thessaloniki, “Georgios Papanikolaou” Hospital, Greece. Inclusion criteria are: patients with large or massive repairable rotator cuff tears combined with SSN neuropathy on the ipsilateral side. Exclusion criteria are: SSN neuropathy of another cause (e.g., brachial plexus neuropathy), other previous surgery performed on the same affected shoulder, and moderate to severe degenerative changes to the ipsilateral glenohumeral joint (rotator cuff arthropathy).

All patients seen in our institution’s shoulder clinic with MRI-confirmed large or massive repairable rotator cuff tears and suspected SSN neuropathy will undergo NC/EMG studies in order to establish a diagnosis of the neuropathy. Patients with no signs of pain, atrophy, or paresthesia over the SSN distribution area, or patients who refuse to have any more diagnostic processes, will not have NC/EMG studies and will not be considered for the study. If the above studies are positive for SSN neuropathy, and the patients meet the inclusion and none of the exclusion criteria, they will be informed about this trial. If they agree to participate in this trial and sign and date the informed consent, then a further preoperative appointment will be given.

An investigator will clinically examine the patients on the second preoperative appointment. A specific form will be created for each one that will contain demographic, preoperative, and postoperative data (Additional file 2: Table S1). All patient forms will additionally be kept in electronic records for easier data analysis. Randomization and allocation of patients to a specific group will be done by the statistician. All patients, the clinical preoperative and follow-up examiner, and the neurologist performing the NC/EMG studies will be blinded to the type of surgery performed.

The first group of this trial will be the control group and will consist of 21 patients; these will undergo arthroscopic repair of the rotator cuff tear. The second group will also consist of 21 patients. They will undergo the procedure as above, along with an arthroscopic release of the SSN at the same stage.

### Details of preoperative and postoperative examinations

All patients’ demographic data (age, gender, occupation, allergies, smoking status, alcohol consumption) and medical history will be recorded preoperatively. Patients will be examined for specific clinical signs such as Hornblower’s sign, the dropping sign, Jobe’s test, Hawkins test, and the lag sign. Shoulder range of motion (ROM) and shoulder power of the rotator cuff muscles of both arms will also be examined.

The Constant-Murley [14], Disabilities of the Arm, Shoulder and Hand (DASH) [15], and American Shoulder and Elbow Surgeons (ASES) [16] scores will be recorded. The Constant-Murley shoulder score has a 100-point scale and is a commonly used outcome measure for assessing the outcomes of the treatment of shoulder disorders. It includes a pain score, functional assessment, and ROM and strength measures (0 worst score, 100 best score). The DASH outcome measure has 30 questions, which are scored from 1 to 5 according to the severity of a patient’s symptoms (0 best score, 100 worst score). The optional modules of this score will not be measured as, according to our previous experience, most patients will not be professional athletes, musicians, or high-demand workers. Also, this will facilitate an easier comparison of scores between patients. The ASES score is another patient-reported questionnaire and has 17 questions. The first seven of these explore the intensity and type of pain up to a total of 50 points and the rest measure functionality of the shoulder and add another 50 points (0 worst score, 100 best score).

### Study procedure

Patients will be randomized on the admission day and allocated to a group, so that they may undergo arthroscopic rotator cuff repair alone, or combined with arthroscopic release of the SSN. Only the operating surgeon, who will be the same for all patients, will be aware of the randomization results and patient allocation list.

All patients are going to be treated according to the established institutional protocol and the operation will follow well-defined standards in order to minimize adverse factors. All operations will be done with the patient in a beach-chair position, under general anesthesia and with a regional (interscalene) anesthetic block for postoperative analgesia. If general anesthesia is contraindicated, then the procedure will be done with the patient awake or mildly sedated and with the regional block as the main anesthetic agent; these patients will be recorded. Prepping will be done as per hospital protocol with aqueous povidone iodine 7.5%, and draping will be the same for all patients. Standard portals will be made for all patients: posterior, anterior, lateral, and anterolateral; a superior portal will additionally be made for patients undergoing release of the superior transverse scapular ligament.

The procedure will aim at fully restoring the rotator cuff to its anatomical footprint. When the cuff is not repairable then these patients will be excluded from the study. If during the operation the surgeon fails to fully repair the cuff and achieves only a partial repair, these patients will be recorded and followed up but separated from the cohort; their outcomes will be compared with those of the rest. All repairs will be done using a transosseous equivalent technique with peek anchors, knotted medially and knotless laterally. Arthroscopic release will follow the technique published by Lafosse et al. [1]. Visualization will be done mostly by the lateral portal and the shaver will mostly work through a lateral-anterolateral portal. The transverse ligament will be dissected via a superior portal, which is created under direct vision and uses a needle to identify the exact location of the portal.

As per the surgical protocol, all patients will also undergo tenotomy of the long head of biceps and acromioplasty with excision of the coracoacromial ligament. Also, excision of the acromioclavicular joint will be performed only if patients complain of pain in that region along with degenerative changes being shown on X-rays, or found intraoperatively. If any further lesions are detected during the operation, the surgeon will proceed to repair them and such incidents will also be recorded. Skin closure will be performed with 3.0 nylon sutures. Nonadhesive dressings and pressure dressings will be applied to the top of the shoulder and patients will be placed in a 30^o^ abduction sling immediately after completion of surgery.

Patients will stay overnight at the hospital. Physiotherapists will advise them as per the physiotherapeutic protocol (Table 2). The abduction sling will remain for the first 6 weeks. Sutures will be removed 10 days after surgery. Outpatient and physiotherapy clinic follow-up will be arranged at 6 weeks, 3 months, and 6 and 12 months postoperatively. Neurology clinics for NC/EMG studies will be arranged at 6 and 12 months postoperatively. Outcome scores will be collected at 6 and 12 months after surgery.

**Table 2:** Brief physiotherapy protocol for postoperative rehabilitation of repaired large/massive rotator cuff tears

Time	Focus	Range of motion	Recommended exercises	Precautions
0–6 weeks	Tissue healing	No passive assisted or active ROM of shoulder allowed apart from table slides	• Restricted table slides after 2nd week to 30° • AROM of elbow, wrist, hand	• No active reaching • Sling at all times
6–12 weeks	PROM, AAROM with transition to AROM	Regain full PROM and AAROM, transition to AROM at 10 weeks	• PROM of shoulder, pendulums, scapular mobility, ball squeeze begin AAROM at 8 weeks • Isometrics and active progression at 10 weeks	• No resisted activity and lifting • Avoid overloading of supraspinatus • Must regain scapular rhythm before transition to strengthening
12–24 weeks	AROM and progressive strengthening	Regain full AROM	• Continue PROM and AAROM as needed • Initiate strengthening and progress AROM to functional planes • Progress proprioceptive drills, rhythmic stabilization, push-ups, resistance band	• No heavy or repetitive overhead activities • Limited return to gym after 20th week if AROM with satisfactory scapular rhythm has been achieved • Free full ROM
24 weeks–1 year	Return to sports and physical activity if ROM and strength adequate		• Progression of strengthening • Continue resistance band, proprioceptive drills and periscapular stabilization until full return to physical activity and sports	• Return to sports • Progress gym lifting

*AAROM* active assisted range of motion, *AROM* active range of motion, *PROM* passive range of motion, *ROM* range of motion

### Procedure risks/complications

Releasing the superior transverse scapular ligament carries the risk of damage to the SSN and artery. Normally, the nerve lies inferior to the ligament and medially is protected by the shaver during soft-tissue resection. The artery, however, passes superior to the ligament and is potentially in jeopardy. Care will be taken not to cut the artery. However, if it is injured, coagulation may be achieved by using a radiofrequency device. No additional morbidity has been observed in such cases due to the rich collateral circulation of this region. Patients will be informed of these risks before signing the Consent Form.

### Trial duration

The main trial period will start 6 weeks prior to surgery (where clinical NC/EMG and MRI evaluation will be done) and will include the operative intervention and the postoperative period up to the last follow-up. Follow-up clinical examinations and NC/EMG studies for all patients will be conducted at 6 and 12 months after surgery. MRI studies for each patient will be done at least 1 year after the date of the operation.

With a recruitment period of 24 months and a follow-up period of 12 months, the total length of this study is expected to be approximately 36 months. This trial will end when all the necessary 42 patients have completed it. Also, the principal investigators may decide to terminate the study at any time for any reasonable or medical reasons. The final report of this trial will follow the CONSORT guidelines and its extension for nonpharmacological interventions.

### Statistics

#### Sample size calculation

Statistical analysis of previous relevant medical literature was used in order to identify the number of patients needed for this trial. Since no similar studies could be found, we searched for results from similar articles relative to the trial. Costouros et al. [7] treated six patients with massive rotator cuff tears and SSN neuropathy by arthroscopically repairing the cuff. They found complete nerve recovery in four out of six patients. Oizumi et al. [17] performed an arthroscopic release in 18 patients with a rotator cuff tear and all had complete recovery from SSN palsy, although the diagnosis was clinical and no NC/EMG studies were done. Lafosse et al. [1] mention unpublished data of 29 massive rotator cuff tear cases accompanied by SSN neuropathy, where no statistical difference was found between patients who underwent nerve release and patients who did not; however, no further data or tables are provided and the cases are anecdotal. Therefore, we considered that 100% of patients undergoing nerve release and 66.67% of patients undergoing rotator cuff repair only could have full nerve recovery.

We used the statistical program G*Power [18] in order to do a priori power analysis of the primary endpoint of this study. By using a Fisher’s exact test to compute differences between these groups, a 100% success probability of the first group (nerve release) and a 66.67% probability of the second group, a error probability set at 0.05, power set at 0.8, and an allocation ratio equal to 1, a total of 38 patients were found to be needed, 19 for each group. Usually, patients who undergo such operations rarely miss standard follow-ups in this institution because these are part of their physiotherapy and clinical postoperative care (apart from the NC/EMG studies). If 10% of recruited patients fail to attend follow-ups for any reason, then 42 patients will be required for this RCT.

#### Statistical methods

Statistical analysis will be carried out after all patients have completed 12 months of follow-up. Fisher’s exact test will be used for the primary endpoint of the trial. In order to analyze secondary endpoints, the type of data distribution will be studied. If the results follow a normal distribution, the chi-square test and *t* test will be used to compare qualitative and quantitative parameters accordingly. If the results do not follow a normal distribution, quantitative parameters will be studied with the Mann-Whitney test. Depending on the data collected, further tests, such as the Wilcoxon and Kruskal-Wallis tests, may be done for subgroups. Statistical differences will be considered significant if *P* < 0.05.

#### Randomization/blinding

The choice of resecting or not the transverse scapular ligament will be done in a randomized manner. Randomization will be made by using the Microsoft Office Excel program and its RAND function. Forty-two cells will be created and the numbers in them will be rounded up so that they have no decimals. Odd numbers will create the first group and even numbers will create the second group. If RAND function fails to create even groups, it will be redone until even groups of odd and even numbers are achieved. Patients will be placed at each consecutive cell at the time of recruitment, after inclusion in this trial, and will be randomized by the statistician. The surgeon will be informed about the type of treatment needed for patients on the admission day. This will be facilitated with opaque envelopes containing the patient’s group, which the primary investigator will have received from the statistician. The primary investigator will place this envelope inside the patient’s physical records just before the patient is taken to the operating room. An operating department practitioner nurse will open the envelope just after the surgeon finishes the rotator cuff repair and will instruct him, according to the patient’s group, whether to release the ligament or not. By doing this, the surgeon will not be biased by knowing the patient’s group before repairing the cuff (avoidance of performance bias).

The surgeon, therefore, clearly cannot be blinded to the type of procedure, but this will not bring any bias to the study. Patients, physiotherapists, and the clinician measuring outcomes will be blinded to each patient’s allocated treatment. The neurologist will also not know the type of surgery performed. This blinding will assist in obtaining objective results while performing NC/EMG studies and should exclude such bias.

### Subject withdrawal

Patients may withdraw from the study at several stages of the trial, if they wish it, even after having signed the Consent Form. They may also reject an intervention or reject a follow-up evaluation. If patients reject the operation, then they will be excluded from the study. If they reject a follow-up evaluation, then their data will be analyzed and individually reported at the final report of this trial, irrespective of their group. During surgery, if moderate or severe rotator cuff arthropathy is found, or if the surgeon fails to repair the cuff or release the SSN, then patients will be withdrawn from the trial. Postoperatively, patients may be removed from the RCT if they deteriorate and a diagnosis of rotator cuff re-rupture is made by ultrasound or MRI studies.

## Discussion

Published studies have demonstrated a relation between SSN neuropathy and large/massive retracted rotator cuff tears [8, 9]. Mallon et al. analyzed the outcomes of eight patients with SSN neuropathy and a massive retracted rotator cuff tear. All these patients also presented with SSN neuropathy that was confirmed by EMG studies. Four of these patients underwent partial repair of the cuff. Two of them showed re-innervation potentials [8]. In the Shi et al. study, 87 patients underwent NC/EMG studies and MRI of their symptomatic shoulder. The study concluded that an exact relation between SSN neuropathy and rotator cuff pathology remains unclear and found that SSN neuropathy is associated with tendon tear size. However, this relation was not linear as minimally retracted tears were more strongly correlated with SSN neuropathy when compared to bigger ones. This study aims to better define that relationship and to discover the most effective treatment for this disease. If both interventions performed in this trial have the same potential in reversing SSN neuropathy, then there will be no need to release the SSN when dealing with SSN neuropathy in the setting of large/massive rotator cuff tears. Just by repairing the tendon and, therefore, the length of the muscle/tendon unit, the nerve will be freed and have potential for re-innervation.

Experimental studies have revealed a possible relation between the size of rotator cuff tear and degree of nerve damage. Warner et al., in their cadaveric study, determined that lateral reduction of rotator cuff tears of more than 3 cm might result in significant tension on the SSN and entrapment as the nerve passes through the transverse scapular ligament [19]. Greiner et al., in another cadaveric study, found that that the maximum lateral advancement of a retracted rotator cuff tear is between 1 and 3 cm. As advancement progresses, the neurovascular pedicle is placed under increased tension [20]. However, Hoellrich et al. did EMG studies and did not find any SSN neuropathy after repairs of massive rotator cuff tears in nine patients (mean advancement of 2.5 cm, range 2.0 to 3.5 cm). They concluded that the tendons could be mobilized and advanced up to 3.5 cm without putting the SSN at risk [21]. Although studies suggest nerve damage even after small traction, the medical literature has shifted towards finding a relation between SSN neuropathy and massive rotator cuff tears (more than a 5-cm tendon gap).

Electromyography studies are generally considered in patients with unexplained shoulder pain, but his has been debated as authors disagree about the sensitivity and specificity of EMG [22–24]. A damaged nerve can produce several abnormal findings on an EMG, such as reduced amplitude of motor potentials, increased spontaneous activity, fibrillations, polyphasic activity, and fibrillation and sharp waves. The accuracy of NC/EMG studies in assessing SSN neuropathy has been challenged, as there are no clear criteria [12]. Diagnosing SSN neuropathy in patients with massive rotator cuff tears can be even more difficult, especially clinically, as the symptoms between these entities overlap. Due to the lack of any other validated foolproof test, however, NC/EMG studies have become the “gold standard” [12].

It has been impossible so far to validate NC/EMG studies in patients with rotator cuff tears. Muscle biopsy or neural cell adhesion molecule histochemistry can indicate denervated muscle as shown in experimental models. However, its accuracy fails in the setting of a tenotomized muscle [25], which also affects EMG studies [26]. Thus, no trials have previously validated NC/EMG studies for the diagnosis of SSN neuropathy in the setting of large/massive rotator cuff tear, nor have they defined concise diagnostic criteria. Colin et al., in an attempt to define the prevalence of suprascapular neuropathy with repairable massive rotator cuff tears in 49 patients below 65 years of age, documented the diagnostic criteria that they used when performing NC/EMG studies [12]. Interestingly, they found a low relation (2%) between SSN neuropathy and massive rotator cuff tears.

Techniques used in order to perform SSN release can be open or arthroscopic. Open SSN release has provided good results in a series of 39 patients; 28 showed improvement of supraspinatus muscle strength to grade 4 [11]. However, arthroscopic techniques have gained momentum, possibly due to being less invasive, having better visualization and having the ability to be combined with repair of the rotator cuff without the need for extra incisions.

The technique of arthroscopic transverse scapular ligament dissection has been described previously [1, 27–29]. Lafosse et al. in 2007 reported excellent outcomes in nine out of ten patients who underwent SSN arthroscopic decompression without rotator cuff repair [28]. Complete normalization of latency in NC/EMG studies was found in seven of these patients. Costouros et al. reported six patients with SSN neuropathy related to massive cuff tear. He found complete improvement in EMG function in four and partial recovery in two patients [7]. In a study by Shah et al., 21/24 (87.5%) patients had deep posterior shoulder pain and NC/EMG findings of SSN denervation. After SSN decompression, 17/24 (71%) of these patients had reduced levels of pain at 9 weeks after surgery and demonstrated improved ASES scores [30]. The authors also hypothesized that there may be a subgroup of patients with negative NC/EMG studies presenting with unexplained posterior shoulder pain and that these patients may respond to SSN decompression.

The studies above show promising results after release of the SSN, but reversal of muscle atrophy is not always observed. Kim et al. reported 90% of patients with profound muscle weakness regaining supraspinatus muscle strength to grade 4 or better, but infraspinatus muscle strength gains were less predictable [11]. Fabre et al. reported 89% of patients obtaining good-to-excellent outcome after open SSN decompression, but reversal of muscle atrophy was only seen in half of these patients (52%) [31].

In conclusion, published studies so far deliver some evidence regarding the relation between SSN and massive cuff tears and suggest good/excellent postoperative outcomes, with or without releasing the SSN. These cohort studies provide ultimately inconsistent and ambivalent results, as they do not measure their current management with an alternate treatment. The need for comparable studies becomes apparent in order to create a guideline, which would assist shoulder surgeons in making effective and safe treatment choices when dealing with patients with repairable large/massive rotator cuff tears and SSN neuropathy.

### Trial status

The study is currently in its recruiting phase.

## Additional files

Additional file 1: SPIRIT checklist: recommended items to address in a clinical trial protocol and related documents*. (PDF 499 kb)
Additional file 2: Table S1. Patient forms which will contain preoperative and postoperative clinical signs and brief outcomes of neurophysiological and imaging studies. (PDF 667 kb)

## Abbreviations

ASES: American Shoulder and Elbow Surgeons
CONSORT: Consolidated Standards of Reporting Trials
DASH: Disabilities of the Arm, Shoulder and Hand
EMG: Electromyography
MRI: Magnetic resonance imaging
NC: Nerve conduction
RCT: Randomized controlled trial
ROM: Range of motion
SPIRIT: Standard Protocol Items: Recommendations for Interventional Trials
SSN: Suprascapular nerve
