# Supplementary material for: Suprascapular neuropathy in the setting of rotator cuff tears: study protocol for a double-blinded randomized controlled trial
Source: Trials. 2016 Nov 22;17:554. doi: 10.1186/s13063-016-1672-y (PMC5120427; doi:10.1186/s13063-016-1672-y)
Supplement: Additional file 2: Table S1. — Patient forms which will contain preoperative and postoperative clinical signs and brief outcomes of neurophysiological and imaging studies. (PDF 667 kb) [file 13063_2016_1672_MOESM2_ESM.pdf]

Table S1: Patient forms which will contain preoperative and postoperative clinical signs, brief outcomes of neurophysiological and imaging studies.

PATIENT FORM Allocation/Number:

INCLUSION DATE:_______ OPERATION DATE:_____

NAME/SURNAME:
GENDER: AGE:

DIAGNOSIS (MENTION SIZE OF CUFF TEAR):

RELATED SHOULDER PROBLEMS (E.G: IMPINGEMENT, A/C JOINT PAIN, INSTABILITY)

DATE OF DIAGNOSIS ESTABLISMENT IF DIFFERENT FROM INCUSION DATE:

**CLINICAL SIGNS**	PREOPERATIVELY	6 MONTHS POST-OP	12 MONTHS POST-OP
HORNBLOWER SIGN			
DROPPING SIGN			
JOBE SIGN			
NEER SIGN			
HAWKIN’S SIGN			
LAG SIGN			
ABDUCTION			
FORWARD ELEVATION			
INTERNAL ROTATION			
EXTERNAL ROTATION			
SUPRASCAPULAR SENSORY FUNCTION (0,1,2), REGIONAL PAIN			
**POWER** SUPRASPINATUS			
SUBSCAPULARIS			
INFRASPINATUS			
TERES MINOR			
ATROPHY			
CONSTANT SCORE			
SPADI SCORE			
ASES SCORE			
DASH SCORE			

OP MRI FINDINGS AND DATE:	PRE- OP NC/EMG FINDINGS AND DATE:
TWELVE MONTHS POST-OP MRI FINDINGS:	SIX MONTHS NC/EMG FINDINGS:
	12 MONTHS NC/EMG FINDINGS:
